# Detection and Management of Elevated Intracranial Pressure in the Treatment of Acute Community-Acquired Bacterial Meningitis: A Systematic Review

**DOI:** 10.1007/s12028-023-01937-5

**Published:** 2024-02-14

**Authors:** Victor Gabriel El-Hajj, Ingrid Pettersson, Maria Gharios, Abdul Karim Ghaith, Mohamad Bydon, Erik Edström, Adrian Elmi-Terander

**Affiliations:** 1 Department of Clinical Neuroscience, Karolinska Institutet, Stockholm, Sweden; 2 Mayo Clinic Neuro-Informatics Laboratory, Mayo Clinic, Rochester, MN USA; 3 Department of Neurological Surgery, Mayo Clinic, Rochester, MN USA; 4 Capio Spine Center Stockholm, Löwenströmska Hospital, Upplands-Väsby, Sweden; 5 Department of Surgical Sciences, Uppsala University, Uppsala, Sweden

**Keywords:** Acute bacterial meningitis, Intracranial pressure monitoring, Intracranial hypertension management, External ventricular drain, Outcomes

## Abstract

Acute bacterial meningitis (ABM) is associated with severe morbidity and mortality. The most prevalent pathogens in community-acquired ABM are *Streptococcus pneumoniae*, *Neisseria meningitidis*, and *Haemophilus influenzae*. Other pathogens may affect specific patient groups, such as newborns, older patients, or immunocompromised patients. It is well established that ABM is associated with elevated intracranial pressure (ICP). However, the role of ICP monitoring and management in the treatment of ABM has been poorly described.An electronic search was performed in four electronic databases: PubMed, Web of Science, Embase, and the Cochrane Library. The search strategy chosen for this review used the following terms: Intracranial Pressure AND (management OR monitoring) AND bacterial meningitis. The search yielded a total of 403 studies, of which 18 were selected for inclusion. Eighteen studies were finally included in this review. Only one study was a randomized controlled trial. All studies employed invasive ICP monitoring techniques, whereas some also relied on assessment of ICP-based on clinical and/or radiological observations. The most commonly used invasive tools were external ventricular drains, which were used both to monitor and treat elevated ICP. Results from the included studies revealed a clear association between elevated ICP and mortality, and possibly improved outcomes when invasive ICP monitoring and management were used. Finally, the review highlights the absence of clear standardized protocols for the monitoring and management of ICP in patients with ABM. This review provides an insight into the role of invasive ICP monitoring and ICP-based management in the treatment of ABM. Despite weak evidence certainty, the present literature points toward enhanced patient outcomes in ABM with the use of treatment strategies aiming to normalize ICP using continuous invasive monitoring and cerebrospinal fluid diversion techniques. Continued research is needed to define when and how to employ these strategies to best improve outcomes in ABM.

**Supplementary Information:**

The online version contains supplementary material available at 10.1007/s12028-023-01937-5.

## Introduction

Acute bacterial meningitis (ABM) is a life-threatening, infectious disease that affects patients of all ages. Even with the best medical treatment [1], it is associated with severe morbidity and mortality [2]. The classical symptoms include fever, headache, neck stiffness, and an altered level of consciousness. More than 95% of patients with ABM will present with at least two of these symptoms [3]. The course of ABM is accompanied by an elevated intracranial pressure (ICP) in up to 93% of patients [4]. The rise in ICP is multifactorial, with major elements being cerebral edema of vasogenic, cytotoxic, or interstitial types [5]. Other mechanisms contributing to elevated ICP include the loss of cerebrovascular autoregulation with increased vasodilation, venous thrombophlebitis producing venous congestion, and the development of hydrocephalus due to impaired cerebrospinal fluid (CSF) circulation and reabsorption [5, 6].

The most feared complication of elevated ICP is brain herniation, which may lead to coma or death [7]. Elevated ICP and its consequences have been described among the primary causes of death in patients with ABM [8–10]. Although it is well known that early diagnosis followed by timely administration of corticosteroids and antibiotics are crucial for the successful management of these patients, there is no consensus on whether to monitor ICP or how to manage ICP elevation in these patients [2].

The current guidelines on the treatment of ABM, issued by the European Society of Clinical Microbiology and Infectious Diseases, recommend a computed tomography (CT) scan on suspicion of elevated ICP and/or intracranial space-occupying lesions (focal neurologic deficits, new-onset seizures, and Glasgow Coma Scale (GCS) of < 10, all indicators of elevated ICP), before performing a lumbar puncture [6]. However, CT scanning cannot accurately assess ICP and lacks temporal resolution [11, 12]. Instead, the use of more accurate invasive ICP monitoring methodologies may be warranted [13, 14]. However, the timing and indications for invasive ICP monitoring in the management of ABM remain unclear [2, 14]. Although some authors suggest that invasive ICP monitoring should be considered in patients with ABM with a GCS score below 8 [14–16], others argue that ICP monitoring should be initiated earlier in the course of the disease to ensure the best neurological outcomes [17]. The guidelines for bacterial central nervous system infections issued by the Swedish Society for Infectious Diseases suggest that invasive ICP monitoring and treatment should be initiated in rapidly deteriorating or comatose patients with an elevated pressure on lumbar puncture [18]. Similarly, the European Society of Clinical Microbiology and Infectious Diseases has recognized the need for further research to establish recommendations for the use of ICP monitoring and ICP-based management approaches in patients with ABM. In line with that, the aim of this systematic review was to investigate the effect of invasive ICP monitoring and management on morbidity and mortality for the treatment of community-acquired ABM.

## Methods

This systematic review is in accordance with the Preferred Reporting Items for Systematic Reviews and Meta-Analyses [19] guidelines (Supplementary file 1, Table S1). The review protocol was registered within the International Prospective Register of Systematic Reviews (PROSPERO) (Registration identifier CRD42022332706. Date of registration 25/05/2022). Our review complies with all ethical guidelines and did not require ethical approval.

### Eligibility Criteria

#### Types of Studies

The systematic review only included peer-reviewed human studies, regardless of the date of publication. Case reports, reviews, editorials, letters, and conference abstracts were excluded.

Studies in the English, French, Swedish, and Norwegian languages were eligible for inclusion.

### Type of Population

Only studies on patients with community-acquired ABM were considered. Studies on viral, cryptococcal, tuberculous meningitis, or iatrogenic meningitis were excluded, with the exception of articles in which these etiologies constituted a minority of the study cohort. When possible, these patients were excluded to better serve the scope of this review.

### Type of Intervention

The aim of this review was to summarize the current evidence on the efficacy of ICP-based management strategies for community-acquired ABM. Only studies reporting the use of a technology, intervention, or treatment for either the detection or the management of elevated ICP in community-acquired ABM were considered.

### Type of Outcome Measures

The main outcomes of interest to this review were morbidity and mortality. Other outcomes included length of hospital stay, ICP-related metrics (including opening ICP, overall mean or median ICP, number of ICP peaks, etc.), and complications related to invasive ICP monitoring or management. Studies without any outcome of interest were excluded.

### Databases and Search Strategy

Articles were selected from four different electronic search engines and databases including PubMed, Web of Science, Embase, and the Cochrane Library. The search strategy used in this review combined the following terms using simple Boolean operators: Intracranial Pressure AND management OR monitor* AND bacterial meningitis (Supplementary file 1, Table S2).

### Study Selection

Searches across all search engines from inception until October 2022, yielded a total of 403 publications. After duplicate removal, the remaining 307 studies were transferred to Rayyan where the selection process took place [20]. The studies were first screened based on titles and abstracts by two independent and blinded reviewers (I.P. and V.G.E.). Then, full-text articles were assessed by three independent and blinded reviewers (V.G.E., A.E.T., and E.E). Inter-reviewer conflicts were resolved through discussion.

### Data Extraction and Synthesis

Data extraction adhered to a predefined extraction template encompassing the following: first author last name, date of publication, study characteristics and design, sample size, ABM diagnosis criteria, control group, ICP monitoring technique, indications for monitoring, ICP management approach, indications for management, and outcomes including patient mortality and morbidity, invasive monitoring-related complications, and posttreatment ICP-related outcome measures.

Extraction was performed by two independent authors (V.G.E, and M.G.), and the two extraction sheets were cross checked by a third blinded and independent author (A.E.T).

Because of the small number and heterogeneity of the studies, including different population types, comparators, devices used, and primary outcome measures, a meta-analysis could not be performed. Instead, we opted for a narrative and qualitative description of the data.

### Risk of Bias and Evidence Certainty Assessment

Risk of bias was assessed using the Newcastle–Ottawa Scale, a scoring system designed for observational studies and allowing a maximum of 9 points per study. Because two of the studies were interventional in nature, the Newcastle–Ottawa Scale could not be used, and the National Institutes of Health quality assessment tool was employed instead. The results of this assessment are provided (Supplementary file 1, Tables S3 and S4). The Grading of Recommendations, Assessment, Development, and Evaluation (GRADE) approach was employed to rate the body of evidence supporting the review’s key findings [21]. A GRADE summary of findings table assembled using the GRADEpro Guideline Development Tool is provided [22].

## Results

### Baseline Characteristics and Risk of Bias Assessment

**Fig. 1:**
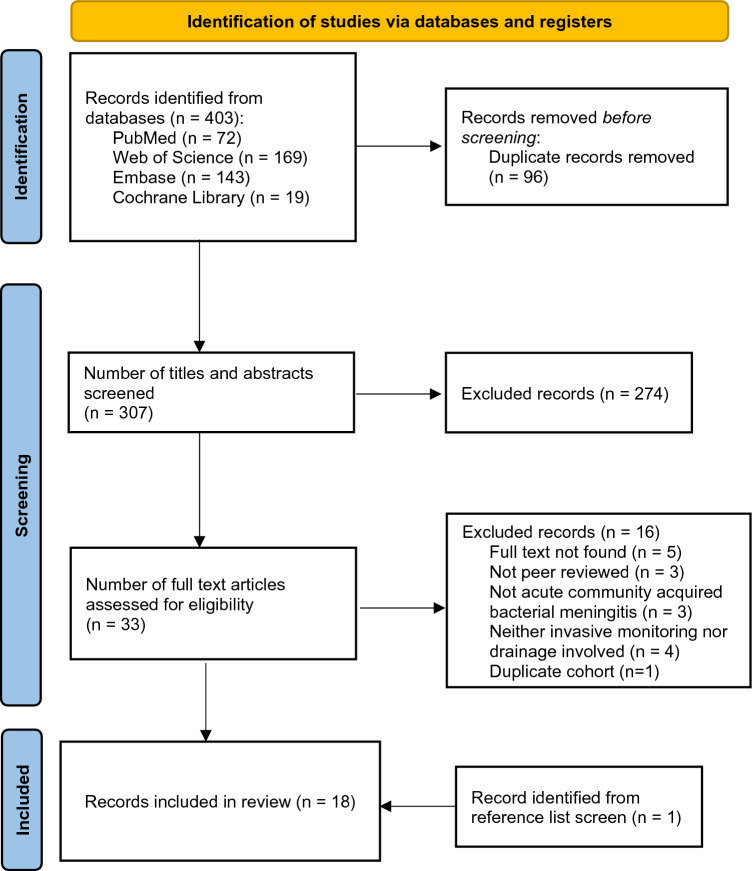
PRISMA 2020 flow diagram for new systematic reviews which included searches of databases, registers, and other sources. PRISMA, Preferred Reporting Items for Systematic Reviews and Meta-Analyses

After initial title and abstract screening, 33 remaining articles were gathered in full-text form. The final screening process resulted in 16 exclusions, leaving a total of 17 studies to be included. After screening of the reference lists of the included studies, one more eligible study was identified, amounting to a total of 18 included studies (Fig. 1).

**Fig. 2:**
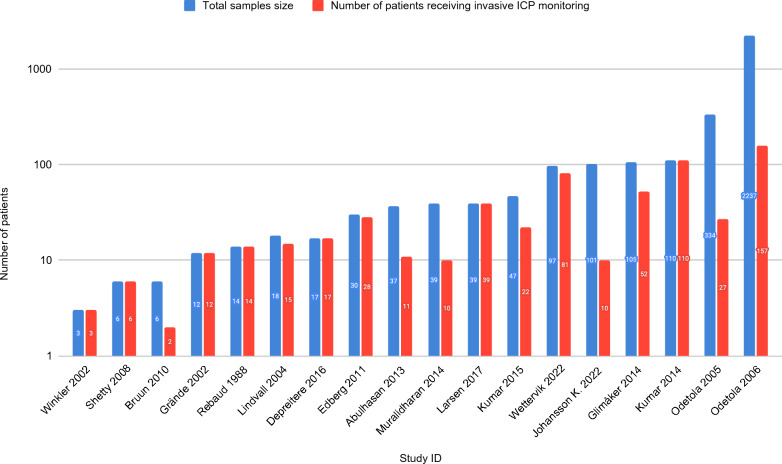
Logarithmic-scale histogram showing the sample size and number of patients treated with invasive monitoring in each of the included studies. ICP, intracranial pressure

Nine retrospective cohort studies, seven case series, and two trials, in which one was randomized, were included in this review (Table 1). The majority of the studies were conducted in Europe (*n* = 11): six studies in Sweden, and one each in Denmark, Norway, Germany, Belgium, and France. Four were performed in North America, and three were performed in India. Sample sizes varied between 3 and 2237 patients (median 38; interquartile range 15–100). Among these, a total of 616 patients (median 16; interquartile range 10–36) received any form of invasive ICP monitoring (Fig. 2). Eight studies targeted the adult population, seven targeted the pediatric population, two studies had mixed populations, and one study was poorly defined. In most studies, the diagnosis of ABM relied on a combination of clinical presentation, CSF analysis, and microbial cultures. Two studies failed to mention how the diagnosis of meningitis was established. Critical appraisal of the studies showed that most had a moderate to high risk of bias, whereas only a few studies had a low risk of bias (*n* = 4).

**Table 1:** Baseline characteristics and bias scores of the included studies

Study ID	Study location	Study design	Age groups	Sample size	Invasive ICP monitoring	Experimental vs. control or comparator groups	Diagnosis of meningitis	Risk of bias
Rebaud [34]	France	Observational	Pediatric	14	14	None	NM	Moderate to high
Grände [21]	Sweden	Observational	Both	12	12	None	NM	Moderate to high
Winkler [16]	Germany	Observational	Adults	3	3	None	Cultures	Moderate
Lindvall [4]	Sweden	Observational	Adults (mostly)	18	15	None	Cultures	Moderate to high
Odetola [42]	United States	Observational	Pediatric	334	27	None	CSF analysis	Low
Odetola [32]	United States	Observational	Pediatric	2237	157	With vs. without ICP monitoring	Cultures	Moderate to high
Shetty [33]	India	Observational	Pediatric	6	6	None	Cultures, CSF analysis, and clinical picture	Moderate to high
Bruun [28]	Norway	Observational	Both	6	2	None	Cultures and clinical picture	Moderate
Edberg [29]	Sweden	Observational	Adults (mostly)	30	28	None	Cultures, CSF analysis, and clinical picture	Moderate
Abulhasan [31]	Canada	Observational	Adults	37	11	With vs. without LD	Cultures, CSF analysis, and clinical picture	Low
Glimåker [9]	Sweden	Interventional	Adults (mostly)	105	52	ICP management vs. control	Cultures, CSF analysis, and clinical picture	Low
Kumar [27]	India	Interventional	Pediatric	110	110	ICP vs. CPP-targeted management	Cultures, CSF analysis, and clinical picture	Low
Muralidharan [26]	United States	Observational	Adults	39	10	None	Cultures, CSF analysis, and clinical picture	Moderate to high
Kumar [30]	India	Observational	Pediatric	47	22	EVD vs. VP shunt vs. antibiotic only	CSF analysis and clinical picture	Moderate to high
Depreitere [17]	Belgium	Observational	Not stated	17	17	None	Cultures	Moderate to high
Larsen [25]	Denmark	Observational	Adults	39	39	None	Cultures, CSF analysis, and clinical picture	Moderate to high
Johansson K. [44]	Sweden	Observational	Pediatric	101	10	None	Cultures, CSF analysis, and clinical picture	Moderate
Wettervik [24]	Sweden	Observational	Adults (mostly)	97	81	None	Cultures, CSF analysis, and clinical picture	Moderate

CSF, cerebrospinal fluid, CPP, cerebral perfusion pressure, EVD, external ventricular drain, Exp, experimental, ICP, intracranial pressure, ID, identifier, LD, lumbar drain, NM, not mentioned, Obs. Observational, VP shunt, ventriculoperitoneal shunt

### ICP Monitoring

Intracranial pressure in patients with ABM was measured using invasive methods in 18 studies, with external ventricular drain (EVD) and intraparenchymal monitoring devices being the most widely used (Table 2). Six and three studies reported the use of either EVD or intraparenchymal monitoring devices, respectively. Seven other studies reported the use of both, with EVD being most often chosen as the first alternative. One study used subdural catheters and another one used lumbar drain for ICP measurement [9].

**Table 2:** The tools used by each study for ICP measurement, estimation, or monitoring

Study ID	Invasive detection of elevated ICP			Indications for invasive ICP monitoring	Main study findings
	EVD	Intraparenchymal monitor	Other		
Rebaud [34]	No	No	Subdural catheter in infants with open fontanel	Severe CNS infections GCS ≤ 7	High ICP and low CPP in meningitis ICP monitoring important in comatose patients
Grände [21]	Yes	No	No	Clinical picture indicative of an elevated ICP: Loss of consciousness, agitation, increase in blood pressure and pupil dilation	ABM is associated with increased ICP, which can be reduced using the Lund Concept
Winkler [16]	Yes	No	No	Comatose patients with ABM	ICP monitoring of patients with meningitis should be considered regardless of the CT appearances
Lindvall [4]	Yes	Yes	No	GCS ≤ 8 or RLS ≥ 3	ICP was higher and CPP decreased in nonsurvivors. Treatment should include neurointensive care and continuous ICP measurement, resembling Lund Concept
Odetola [42]	Yes	No	No	Subjective decision by the doctor in charge	There was significant variation in the use of ICP monitors among the various PICUs without statistical association with survival
Odetola [32]	Yes	No	No	NM	The use of ICP monitoring was not statistically associated with mortality
Shetty [33]	No	Yes	No	GCS < 7, evidence of elevated ICP on CT or MRI	Reduced ICP and a CPP > 50 mm Hg was associated with survival
Bruun [28]	No	Yes	Lumbar drain	Doctor’s decision during neurosurgical procedure	Patients with early signs of intracranial complications should be transferred to a hospital with neurosurgical services
Edberg [29]	Yes	Yes	No	RLS: ≥ 3B GCS ≤ 8 (However, in cases of rapid deterioration it was initiated earlier)	Patients with ABM should be admitted to neurointensive care units for ICP monitoring and management according to modern neurointensive care principles
Abulhasan [31]	No	No	Lumbar drain	No standardized treatment protocols	The use of lumbar drainage in ABM contributed to low mortality and morbidity
Glimåker [9]	Yes	Yes	No	GCS ≤ 9 or 10 if lumbar pressure > 400 mm H_2_O RLS ≥ 3	ICP-targeted therapy, mainly CSF drainage, reduces mortality and improves the overall outcome
Kumar [27]	No	Yes	No	GCS ≤ 8 Clinical and radiological signs	CPP-targeted therapy was superior to ICP-targeted therapy in ABM by reducing mortality and morbidity
Muralidharan [26]	Yes	Yes	No	GCS ≤ 8	High mortality and morbidity in patients with increased ICP
Kumar [30]	Yes	No	No	NM	EVD may improve cure rate and hasten clearing of CSF infection
Depreitere [17]	Yes	No	No	GCS (median 8)	Significant correlations between outcome and highest ICP, number of episodes when CPP < 50 mm Hg and lowest CPP. Treatment was influenced by ICP in all patients
Larsen [25]	Yes	Yes	No	GCS < 9 or signs of elevated ICP on CT	ICP monitoring should be used in all ABM cases with a GCS < 9 regardless of brain CT findings
Johansson (2020)	Yes	Yes	No	MeningiSSS > 6	The MeningiSSS is a helpful score for decisions concerning ICP management
Wettervik [24]	Yes	Yes	No	GCS < 8, sudden clinical deterioration, repeated seizures, severe psychomotor agitation, clinical signs of brain herniation, radiological findings	CSF drainage was often sufficient to control ICP. Clinical outcome was better than in earlier studies

AMB: acute bacterial meningitis, CNS: central nervous system, CPP: cerebral perfusion pressure, CT: computed tomography, EVD: external ventricular drain, GCS: Glasgow Coma Scale, ICP: intracranial pressure, ID: identifier, MeningiSSS: xxx, MRI: magnetic resonance imaging, NM: not mentioned, PICU: xxx, RLS: Reaction Level Scale

Management of patients in need of invasive ICP monitoring is typically performed at an intensive care unit. In studies in which this information was available (13/18), a deteriorating neurological status was the main indication. In some studies (11/18), authors used standardized and objective metrics such as the GCS, or the Reaction Level Scale. In other studies, different surrogates were used, such as clinical and radiological signs or perceived severity of the illness (7/18). The GCS and Reaction Level Scale thresholds for initiation of ICP monitoring were generally set to below 7–9 or above 3, respectively (Table 2).

All six studies that reported the use of CT in conjunction with invasive techniques confirmed that CT was less reliable and could severely underestimate a raised ICP [4, 16, 17, 23–25]. Muralidhar et al. [26] found that once abnormal head CT findings were detected, clinical outcomes were remarkably worse. Similar findings were noted by Wettervik et al. [24], who found a significant association between both compression of the basal cisterns on admission CT and pupillary abnormalities and unfavorable outcomes or death. Wettervik et al. [24] did not find any association between elevated ICP and positive CT findings, which they attributed to the effective early detection and management of elevated ICP before the development of radiological signs.

### Management of Elevated ICP

Intracranial pressure or cerebral perfusion pressure (CPP)-targeted management protocols were implemented in all studies. ICP in combination with CPP-targeted management was used in six studies, whereas the rest mainly involved ICP-centered approaches. Studies applying CPP-targeted management used fluid resuscitation as well as vasopressors to maintain CPP by increasing the mean arterial pressure. Studies focusing on ICP-targeted management employed different combinations of ICP-lowering strategies, including osmotherapy, CSF drainage, thiopental, and decompressive craniotomy. Only the study by Kumar et al. [27] presented a thorough comparison of the two strategies in a randomized controlled trial. In that study, CPP-targeted management was found to be significantly superior to ICP-targeted management in terms of the 90-day mortality (*p* = 0.020).

The ICP-lowering treatments were diverse and included invasive procedures (18/18 studies), hyperventilation (6/18 studies), thiopental and sedation (8/18 studies), osmotherapy typically with mannitol (3/18 studies), the Lund concept (2/18 studies), and hypothermia (1/18 studies). The invasive procedures included EVD (13/18), decompressive craniotomy (4/18), lumbar drain (LD) (3/18), and ventriculoperitoneal (VP) shunt (1/18).

Overall, the studies lacked detailed and standardized treatment protocols. There was typically an incremental and stepwise implementation of the treatments. Hyperventilation and osmotherapy were often used early, whereas thiopental and decompressive surgery were last resorts for the treatment of refractory rises in ICP. The most common indication for CSF drainage was the detection of an elevated ICP, whereas for decompressive surgery, an elevated ICP refractory to all other treatments was usually a prerequisite [17, 24, 28, 29]. In three studies, CSF drainage was used on acute neurological deterioration, which suggested impending cerebral herniation. Details on the indications as presented in each study are presented in Table 3. Complications associated with the use of invasive procedures were seldom reported. EVD-related adverse events, mainly composed of central nervous system infections, were reported in six patients from two different studies [27, 30]. Both studies were conducted at the same institution in India.

**Table 3:** ICP management strategies that were employed in the included studies

Study ID	Was ICP managed?	Goal of ICP management	ICP vs. CPP-targeted management protocol	Management strategies							Indications for invasive procedures
				Mannitol	Hyperventilation	Thiopental and sedation	Hypothermia	Lund concept	Invasive procedures	Invasive techniques used	
Winkler [16]	Yes (in 2/3)	NM	ICP-targeted	Yes	Yes	Yes	Yes	No	Yes	EVD	Clinical signs of cerebral herniation in the form of neurologic deterioration
Lindvall [4]	Yes (in 13/15)	ICP: < 20 mm Hg	ICP and CPP-targeted	No	No	No	No	Yes	Yes	EVD IPM	In case an acute drop in ICP was deemed necessary
Odetola [32]	Yes	NM	Unclear	No	No	No	No	No	Yes	EVD	In case of elevated ICP
Bruun [28]	Yes	NM	ICP-targeted	Yes	Yes	No	No	No	Yes	LD, IPM DC	High ICP for LD For DC: after radiological diagnosis of subdural effusion with associated neurological deterioration
Edberg [29]	Yes	ICP: ≤ 20 mm Hg CPP: > 60 mm Hg	ICP-targeted	No	Yes	Yes	No	No	Yes	EVD IPM DC	For EVD: In case of elevated ICP For DC: In case of elevated ICP despite previous treatment attempts
Abulhasan [31]	Yes	NM	ICP-targeted	No	No	No	No	No	Yes	LD	In case of severe neurological deficits or deterioration and elevated ICP despite previous treatment attempts
Kumar [27]	Yes	ICP group: ICP > 20 mm Hg CPP group: CPP ≥ 60 mm Hg	Group 1: ICP-targeted Group 2: CPP-targeted	Yes	Yes	Yes	No	No	Yes	IPM	In case of elevated ICP
Muralidharan [26]	Yes	NM	Unclear	No	No	No	No	No	Yes	EVD IPM	In case of elevated ICP
Glimåker [9]	Yes	ICP: < 20 mm Hg CPP: > 50 mm Hg	ICP-targeted	Yes	Yes	Yes	Yes	No	Yes	EVD IPM	In case of elevated ICP
Kumar [30]	Yes	NM	ICP-targeted	No	No	No	No	No	Yes	EVD VP shunt	In case of elevated ICP
Depreitere [17]	Yes	To treat associated hydrocephalus	ICP-targeted	No	No	Yes	No	No	Yes	EVD DC	For both EVD and DC: In case of elevated ICP
Larsen [25]	Yes (in 29/39)	ICP: < 20 mm Hg CPP: > 60 mm Hg	ICP and CPP-targeted	Yes	Yes	Yes	No	No	Yes	EVD IPM LD	In case of elevated ICP
Johansson K. (2020)	Yes	NM	Unclear	No	No	No	No	No	Yes	EVD IPM	In case of elevated ICP
Wettervik [24]	Yes	ICP: ≤ 20 mm Hg CPP: ≥ 60 mm Hg	ICP and CPP-targeted	No	Yes	Yes	No	No	Yes	EVD IPM DC	For EVD: In case of elevated ICP For DC: In case of elevated ICP despite previous treatment attempts
Rebaud [34]	Yes	ICP: < 15 mm Hg CPP: > 40 mm Hg	ICP-targeted	No	Yes	Yes	No	No	No	Subdural catheter	Comatose patients
Grände (2002)	Yes	ICP: < 25–30 mm Hg	ICP and CPP-targeted	No	No	No	No	Yes	No	EVD	In case of elevated ICP
Odetola [42]	Unclear	NM	Unclear	N/a	N/a	N/a	N/a	N/a	N/a	EVD	In case of elevated ICP
Shetty [33]	Yes	CPP: > 70 mm Hg in children > 2 y. of age and > 60 mm Hg in children < 2 y	ICP and CPP-targeted	Yes	No	No	No	No	No	IPM	In case of elevated ICP

CPP: cerebral perfusion pressure, CSF: cerebrospinal fluid, DC: decompressive craniotomy, EVD: external ventricular drain, ICP: intracranial pressure, ID: identifier, IPM: intraparenchymal monitor, LD: lumbar drain, N/a: not applicable, NM: not mentioned, VP shunt: ventriculoperitoneal shunt

### Patient Outcomes

#### Length of Hospital Stay

The average length of hospital stay was reported in seven studies and varied from 6 to 32 days. In two studies, shorter hospital stays were associated with the placement of an LD as compared with no LD (14 vs. 17 days; *p* = 0.25) [31] and CPP-targeted management as compared with ICP-targeted management (13 vs. 18 days; *p* = 0.002), of which only the latter was significant [27]. In another study, the authors found that patients receiving ICP monitoring had significantly longer hospital stays (*p* = 0.010), even after propensity score matching of the cohorts [32]. Moreover, hospital stay tended to be longer in pediatric studies compared with adults. The average hospital stay ranged between 6 and 17 days in adult studies and between 13 and 32 days in pediatric studies.

### ICP Outcomes

Intracranial pressure values were quantitatively reported in 15 studies, in which only five adopted a longitudinal approach and also presented follow-up values post ICP treatment (Table 4). One of these five studies randomly assigned patients to either ICP or CPP-targeted management plans and found a decrease in mean ICP in both groups by an average of 9 and 15 mm Hg, respectively [27]. There was a significant reduction in the percentage of patients with ICP elevation from 100 to 0% in a study using the Lund concept [23], and from 28 to 9% in a study using a stepwise management strategy combining hyperventilation, CSF drainage, and thiopental [24]. In a fourth study, a management strategy including thiopental, mechanical hyperventilation, EVD, and hypothermia had reportedly failed in one of two patients [16]. Treatment failure was accompanied by death of one of the patients, whereas the second patient, in whom treatment had shown effect, recovered. The last study recorded a trend of normalizing ICP in patients who had received ICP-lowering therapy according to the Lund concept. This trend was especially notable in patients who had survived compared with nonsurvivors (61.2 vs. 19.4 mm Hg; *p* = 0.001) [4].

**Table 4:** Length of hospital stay, posttreatment ICP and CPP, mortality, and morbidity

Study ID	Maximal level of care	Length of hospital stay	Post treatment		Mortality	Morbidity
			ICP	CPP		
Rebaud [34]	NM	NM	NM	NM	30.8%	33.33%
Winkler [16]	ICU	NM	Of the 2 patients who received ICP lowering treatment, only 1 responded and witnessed a progressive decrease in ICP	NM	66.7%	100.00%
Grände [23]	ICU	NM	No further ICP rises were witnessed after start of the treatment, and ICP fell gradually until normalization in almost all	NM	16.7%	30.00%
Lindvall [4]	ICU	Mean hospital stay: 6.6 days	There was a linear downwards trend of ICP with time in survivors following the treatment plan, which remarkably differed from nonsurvivors in whom the ICP tended to remain elevated	NM	33%	NM
Odetola [42]	ICU	Median ICU length of stay: 2 days (IQR: 1–4)	NM	NM	26%	NM
Odetola [32]	NM	Length of hospital stay was significantly longer in patients who had received ICP monitoring (p < 0.05)	NM	NM	19%	NM
Shetty [33]	NM	NM	NM	*Within the first 48 h:* 3 had stable CPP (no acute drop) 1 had only up to 2 acute decreases in CPP 1 had 3 or more acute decreases in CPP	16.67%	60.00%
Bruun [28]	ICU	Mean hospital stay: 24 days (range: 5–37) Mean ICU stay: 2.5 days (range: 0–9)	NM	NM	16.67%	60.00%
Edberg [29]	ICU	Mean hospital stay: 8 days	NM	NM	31%	NM
Abulhasan [31]	ICU	*Mean overall hospital stay*: Non-LD group: 17 ± 16 days LD group: 14 ± 6 days (p ≥ 0.05) *Mean Neuro-ICU stay:* Non-LD group: 7 ± 4.1 days LD group: 5 ± 4.86 days (*p* ≥ 0.05)	NM	NM	LD group: 0% Non-LD group: 15% (*p* = 0.0001)	NM
Kumar [27]	ICU	*Mean hospital stay:* ICP group: 18 days (range: 14.5–21.5) CPP group: 13 days (range: 10.8–15.2) (*p* = 0.002)	*Mean change in ICP from baseline to 72 h:* ICP group: –9 ± 1.2 mm Hg CPP group: –15 ± 1.2 mm Hg (*p* < 0.001)	*Mean change in CPP from baseline to 72 h:* ICP group: 4 ± 1.9 mm Hg CPP group: 18 ± 1.8 mm Hg (*p* < 0.001)	*During ICU stay*: ICP group: 36.4% CPP group: 18.2% (*p* = 0.032) *Total at 90 days follow-up:* ICP group: 38.2% CPP group: 18.2% (*p* = 0.020)	*Neurological deficits at discharge from ICU:* ICP group: 82.9% CPP group: 53.3% (*p* = 0.005) *Neurological deficits 90 days after discharge:* ICP group: 70.6% CPP group: 37.8% (*p* = 0.004)
Muralidharan [26]	ICU	NM	NM	NM	41%	17%
Glimåker [9]	ICU	NM	NM	NM	Intervention group: 10% Control group: 30% (p < 0.05)	Intervention group: 46% Control group: 68% (p < 0.05)
Kumar [30]	NM	Mean hospital stay: 31.72 days (range: 5–90)	NM	NM	Antibiotic only group: 20% EVD group: 23% VP shunt group: 10%	53.3% at follow-up
Depreitere [17]	ICU	NM	NM	NM	29.4%	NM
Larsen [25]	NM	NM	NM	NM	33.3%	84.60%
Johansson [44]	ICU	Mean hospital stay: 14 days	NM	NM	6%	Short-term sequalae: 22% Long term ones: 16%
Wettervik [24]	ICU	Median ICU stay: 7 days (IQR: 4–12)	On day 3: the ICP was still elevated in 9% of patients (*n* = 7)	On day 3: the CPP was still low in 11% of patients only (*n* = 8)	*Among different groups:* With monitoring: 6.2% Without monitoring: 12% Patients who received last-tier treatment: 80% *Total*: 7%	18%

CPP: cerebral perfusion pressure, CSF: cerebrospinal fluid, EVD: external ventricular drain, ICP: intracranial pressure, ICU: intensive care unit, ID: identifier, IQR: interquartile range, LD: lumbar drain, NM: not mentioned, VP shunt: ventriculoperitoneal shunt

### Morbidity

The morbidity rate was reported in 12 studies and varied between 16 and 100%. Eleven of these studies had further information regarding the nature of the complications, mostly hearing loss, neurological deficits, and headaches. In one study, authors recorded a significant reduction in morbidity with ICP monitoring and CSF drainage added to the treatment strategy compared with the control group, which was not monitored (46 vs. 68%; *p* < 0.05) [9]. A randomized trial comparing ICP and CPP-targeted management found the latter to substantially decrease the risks of both hearing loss (8.9 vs. 37.1%; *p* = 0.005) and neurological deficits (53.3 vs. 82.9%; *p* = 0.005), an effect that persisted at the 90-day follow-up (37.8 vs. 70.6%; *p* = 0.004) [27].

### Mortality

Mortality data were reported in all studies and ranged from 0 to 67%. Abulhasan et al. [31] reported zero mortality in a retrospective cohort of 11 adult patients, in whom either EVD or intraparenchymal ICP monitoring was used. The patients were treated according to an ICP-targeted management strategy in which CSF diversion through LD was the primary treatment. In the comparison group in which LD was not used, a mortality of 15% was seen (*p* = 0.0001) [31]. This was despite the fact that worse admission neurologic scores were found among patients receiving management with LD. Control or comparison groups were also present in four other studies. One of the studies, a nonrandomized trial, showed a significant reduction in the mortality of patients with ABM in their intervention group compared with the controls (10 vs. 30%; *p* < 0.05). In addition to the standard treatment given to both groups, the intervention group had ICP monitoring, and CSF drainage as needed to maintain ICP below 20 mm Hg and CPP more than 50 mm Hg [9]. Another study comparing patient mortality found a lower mortality rate in patients with ICP monitoring compared with controls (6.2 vs. 12%). Monitoring led to CSF drainage in almost half of the patients. In the study, the authors also reported a mortality rate of 80% among patients having received last-tier treatment, including thiopental and decompressive craniectomy [24]. Additionally, one study assessed the benefit of CSF drainage through either EVD or VP shunt compared with control patients who had only standard antibiotic treatment. Their results suggested modest improvements in patient outcomes for the CSF drainage groups, especially through VP shunt [30]. The last study, a randomized controlled trial of pediatric ABM cases that compared ICP and CPP-targeted management found the latter to be associated with a lower mortality (18.2 vs. 38.2%; *p* = 0.02) [27].

In two studies adopting the Lund concept for the treatment of 12 and 15 patients, respectively, the mortality rates were 16.7% [23] and 33% [4], respectively. In the latter [4], it was observed that patients who did not receive or did not respond to treatment with the Lund concept were more likely to die.

ICP and CPP values of survivors could be contrasted with those of nonsurvivors in seven studies in which this information was present [23–25, 27, 33, 34]. Finally, six studies reported higher ICP and/or lower CPP values in nonsurvivors compared with survivors [4, 9, 23, 27, 33, 34].

### Evidence Certainty

The GRADE approach was used to assess the certainty of the body of evidence associated with the main findings in this review (Table 5).

**Table 5:** Narrative GRADE evidence summary table

№ of studies	Certainty assessment						Impact	Certainty	Importance
	Study design	Risk of bias	Inconsistency	Indirectness	Imprecision	Other considerations			
In patients with ABM, ICP measurements were higher, and/or CPP lower, among nonsurvivors as compared to survivors									
6	Observational and interventional studies	Serious	Not serious	Not serious	Very serious^a,b^	Large differences	There seems to be evidence correlating high ICP and/or low CPP to mortality in patients with ABM	⊕⊕⊕◯ Moderate	Critical
Invasive ICP monitoring and ICP management may reduce mortality in selected cases of ABM									
5	Observational and interventional studies	Serious	Not serious	Not serious	Very serious^a,^	None	There is evidence of low certainty suggesting a survival benefit with treatment using invasive ICP monitoring and ICP management, when indicated	⊕⊕◯◯ Low	Important
Invasive ICP monitoring and ICP management may reduce morbidity in selected cases of ABM									
1	Interventional study	Not serious	Not applicable^c^	Not serious	Very serious^a,^	None	There is evidence from one interventional study with a low risk of bias, suggesting reduced morbidity with treatment using invasive ICP monitoring and ICP management, when indicated	⊕◯◯◯ Very low	Important

ABM: acute bacterial meningitis, CPP: cerebral perfusion pressure, GRADE: Grading of Recommendations, Assessment: Development, and Evaluation: ICP, intracranial pressure

^a^Relatively few patients and few events were considered in the analysis

^b^Few studies were considered in the analysis

^c^Not applicable since only one study was involved

## Discussion

This systematic review gathered published evidence on different ICP monitoring and management strategies in community-acquired ABM. ICP monitoring in ABM is of great importance because a significant proportion of patients will develop elevated ICP, and mortality in these patients has repeatedly been correlated to intracranial hypertension. These correlations rely on higher ICP values among nonsurvivors [23–25, 27, 33, 34] or autopsy findings, such as uncal or cerebellar herniation indicative of elevated ICP [9, 10, 16, 24, 31, 35].

### Methods to Detect Elevated ICP

In this review, most authors argued against the use of CT to rule out ICP elevation [4, 16, 17, 23–25]. One study showed that an elevated ICP with visible head CT changes correlated with unfavorable outcomes [26]. CT findings have previously been associated with late stages and end stages of the clinical course in ABM and may consequently be of limited use in improving outcomes [11, 24].

Currently, the mainstay of ICP monitoring relies on invasive measuring devices because of their established superiority to noninvasive alternatives [13, 36–38]. The disadvantages associated with invasive ICP measurement may include availability issues, contraindications [29, 39], and associated risks, such as hemorrhages, iatrogenic central nervous system infections [40], and brain herniation [41].

As one study pointed out, the fear of potential adverse events resulting from the use of invasive techniques may be delaying their use in clinical practice [42]. This reasoning may defeat the purpose of such devices, as their associated benefits often result from early detection of abnormally elevated ICP. Because of the heterogeneity between the included studies, any quantitative analysis comparing the different ICP monitoring strategies for ABM would be inappropriate. However, EVD remains the gold standard for ICP monitoring and may hence be considered in the management of ABM, when indicated [14, 17, 24].

### ICP Management

In this review, CSF drainage using an EVD was the most common strategy for the treatment of elevated ICP, but the use of LD or VP shunts was also reported [25, 28, 31]. Adverse events directly related to the use of these strategies were seldom reported [27, 30]. The reviewed literature clearly indicates the lack of a standardized protocol for the detection and management of elevated ICP in ABM. Most of the strategies were either based on an arbitrary combination of treatments or a tier-based scheme that often differed between studies. The granularity of the published data did not allow a thorough comparison of different strategies, and consequently conclusions regarding the relative efficacy of different treatments could not be made. For instance, CPP-targeted management was directly compared with ICP-targeted therapy only in a pediatric population, in which it was superior in terms of the 90-day mortality. Nonetheless, based on the few comparative studies, some points can be highlighted:

1. In the adult population, CSF drainage through an EVD in addition to conventional therapy was superior to conventional therapy alone, reducing both morbidity and mortality [9].
2. In the adult population, CSF drainage through an LD in addition to conventional therapy was superior to conventional therapy alone, reducing both morbidity and mortality [31].
3. In the adult population, CSF drainage through a VP shunt in addition to conventional therapy showed modest improvements in terms of patient outcomes [30].
4. In a pediatric population, CPP-targeted management with vasopressors was superior to ICP-targeted management based on fluids, osmotherapy, and hyperventilation, without CSF drainage [26].

Overall, findings from several studies highlight the potential mortality benefits of ICP management in patients with severe ABM [9, 24, 31].

In summary, although weak, the evidence points toward certain advantages with the use of invasive ICP monitoring and ICP-based treatment approaches in conjunction with conventional treatment approaches, in selected cases of ABM.

## Limitations

The limitations of this review mainly derive from the inherent limitations of the included articles. Namely, many of the included studies had small sample sizes, intermediate to high risks of bias, and observational study designs, with most being retrospective cohort or case series. Only two studies were interventional, and only one was randomized. A second limitation resides in the heterogeneity of study designs, including both parallel and sequential designs, interventional and observational studies, as well as different inclusion criteria and management approaches. Additionally, the primary end points also differed between the studies. Consequently, the heterogeneity of the available data precluded a quantitative meta-analysis and limited the generalizability of the results. In addition, most of the studies failed to report inclusion and exclusion criteria, which may limit external validity of the results. Finally, only studies in the English, French, Swedish, and Norwegian languages were screened for inclusion, which may also hamper the representativity and generalizability of the results.

### Future Perspectives

Several of the studies in this review concluded the need for randomized controlled trials in determining the role of ICP monitoring and management in the treatment of ABM. At this point, however, we find that a benefit of ICP-based management in ABM has been suggested and that careful ethical considerations must precede launching new randomized controlled trials. Randomly assigning patients to different ICP-based management strategies may be an alternative. In addition, useful information may still be derived from observational studies with well-defined inclusion criteria, management plans, and standardized outcomes measures. It remains to be elucidated at which time point invasive monitoring and ICP-based management should be initiated to provide the greatest benefit. Most of the included studies had initiated treatment in comatose patients. However, it is possible that better outcomes could be achieved with earlier intervention. Finally, although EVD insertion was mainly sought for ICP monitoring or CSF diversion, novel evidence may extend its use toward CSF biomarker tracking [43].

**Fig. 3:**
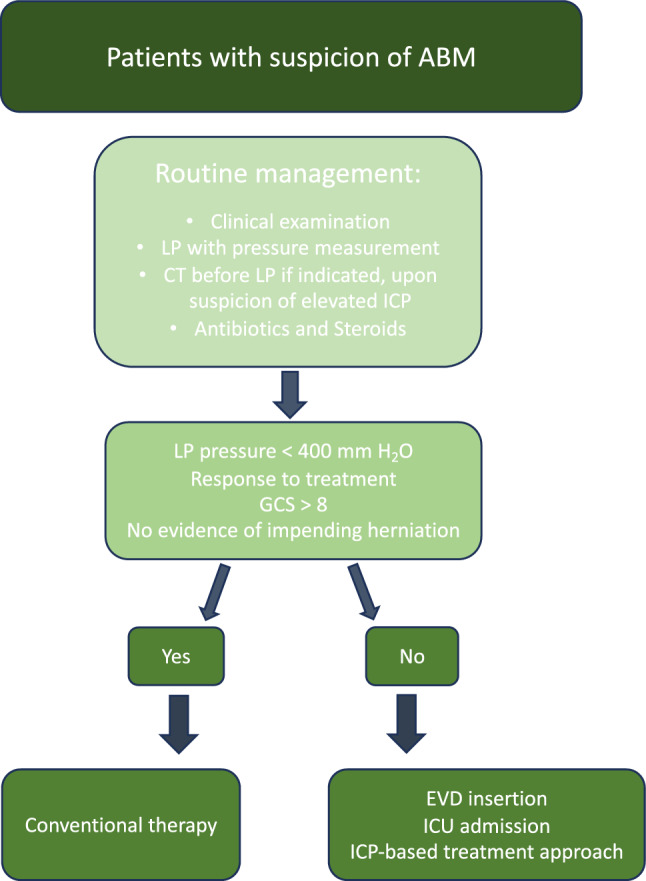
Suggested management of patients with suspicion of ABM based on available guidelines, with the addition of indications for invasive ICP monitoring and ICP-targeted therapy (based on low certainty evidence). ABM, acute bacterial meningitis, CT, computed tomography, EVD, external ventricular drain, GCS, Glasgow Coma Scale, ICP, intracranial pressure, ICU, intensive care unit, LP, lumbar puncture

Based on the findings of this review, we identified the need for more data to support evidence-based guidelines with a structured approach to the use of invasive ICP management strategies in community-acquired ABM [44]. Relying on the study findings, clinical experience, and the guidelines issued by the Swedish Society for Infectious Diseases [18], a strategy is suggested in which all patients with neurological deterioration and elevated lumbar pressure should be treated with invasive ICP monitoring (Fig. 3).

## Conclusions

This review provides insight into the role of invasive ICP monitoring and ICP-based management in the treatment of ABM. The data highlight the association between elevated ICP and mortality, and considerably higher ICP values are found in nonsurvivors compared with survivors. The available evidence is of limited quality but points toward enhanced patient outcomes in community-acquired ABM, with the use of a treatment strategy aiming to normalize ICP using continuous invasive monitoring and CSF diversion techniques (Fig. 3). This is relevant in the most severely affected patients with evidence of elevated ICP who deteriorate despite standard treatment with antibiotics and corticosteroids. Continued research efforts through high quality studies are crucial to determine when and how to employ these strategies to improve outcomes in ABM.

### Supplementary Information

Below is the link to the electronic supplementary material.

Supplementary file1 (DOCX 29 kb)
